# Thyrotoxic Crisis and Disseminated Intravascular Coagulation (DIC) in a Healthy Young Patient Secondary to Sepsis, Presenting With Hyperpyrexia

**DOI:** 10.7759/cureus.84451

**Published:** 2025-05-20

**Authors:** Karthick Raman, Lavanya V

**Affiliations:** 1 Department of Emergency Medicine, Sri Ramakrishna Hospital, Coimbatore, IND

**Keywords:** burch-wartofsky point scale, disseminated intravascular coagulation, hyperpyrexia, sepsis, thyrotoxic crisis

## Abstract

Thyrotoxic crisis is an acute, rare, and potentially fatal complication of hyperthyroidism, associated with significant mortality and morbidity. Its occurrence is almost always precipitated by secondary triggers, and it can arise in both previously diagnosed and undiagnosed cases of hyperthyroidism. According to studies, nearly half of the patients presenting with thyrotoxic crisis had no prior diagnosis of thyroid disorder. Recognizing hyperpyrexia as a key presenting feature can be challenging for emergency physicians, as the initial focus is usually on stabilizing the patient rather than considering endocrine causes early in the evaluation. In view of the complications associated with thyrotoxic crisis, disseminated intravascular coagulation (DIC) is considered a rare but recognized manifestation. In this report, we present a case of a 39-year-old man who arrived at the emergency department with hyperpyrexia, altered mental status, and dyspnea. Initially, his airway was protected, stabilized, and managed with antipyretics, crystalloids, and external cooling measures. He also had features of sepsis and was admitted to the intensive care unit. Subsequently, he developed DIC, received appropriate treatment, and eventually made a full recovery. Since the diagnosis of thyrotoxic crisis is primarily based on clinical judgment, emergency physicians should consider it in the differential diagnosis of patients presenting with hyperpyrexia of unclear etiology. Although DIC is recognized as a rare complication of thyrotoxic crisis, the presence of coexisting sepsis made it challenging to determine whether the thyrotoxic crisis alone was the underlying cause. Therefore, we emphasize the need for further studies to clarify whether DIC in such scenarios arises from thyrotoxic crisis, sepsis, or a combination of both.

## Introduction

Thyrotoxic crisis, also known as thyroid storm, is an endocrine emergency that occurs as a rare, potentially fatal complication of hyperthyroidism and presents with sudden multiorgan involvement [1]. Hyperthyroidism is characterized by excessive thyroid hormone production, most commonly due to Graves’ disease, toxic multinodular goiter, or toxic adenoma [2]. Globally, despite treatment, the associated mortality rate ranges from 8% to 25% [1], and in untreated patients, it can be as high as 100% [3]. The reported mortality rate in India is approximately 8.6% [3]. Therefore, timely identification and appropriate intervention are crucial for improving prognosis [1]. Thyrotoxic crisis is often precipitated by secondary factors such as infections, surgery, trauma, or abrupt discontinuation of antithyroid medications [1].

The clinical presentation of thyrotoxic crisis typically includes hyperpyrexia, neurological manifestations, cardiovascular involvement, and gastrointestinal disturbances [1]. The diagnosis of thyrotoxic crisis is primarily based on clinical judgment and can be supported by established scoring systems such as the Burch-Wartofsky Point Scale and the Japanese Thyroid Association scoring system [1,4]. Therefore, the treating physician should not wait for thyroid hormone levels to initiate treatment [1]. Since multiple complications can arise from untreated thyrotoxic crisis, disseminated intravascular coagulation (DIC) is recognized as a very rare manifestation. Some studies suggest that thyroid hormones may affect coagulation pathways, potentially contributing to the development of DIC [5].

As previously mentioned, early recognition of thyrotoxic crisis significantly improves patient outcomes. It is important for emergency physicians to consider thyrotoxic crisis as a differential diagnosis in patients presenting with the key feature of hyperpyrexia and other associated features suggestive of thyrotoxic crisis, assessed by the established scoring system. Since initial efforts often focus on patient stabilization, maintaining awareness of this rare but life-threatening condition can facilitate prompt diagnosis and early initiation of appropriate treatment, thereby improving outcomes [6]. DIC is a well-documented complication of sepsis; however, its association with thyrotoxic crisis as an isolated cause is not yet clearly understood. Additionally, our patient presented with features of sepsis, making it difficult to determine the exact cause. Further studies are required to delineate better the relationship between thyrotoxic crisis, sepsis, and DIC.

## Case presentation

A 39-year-old male patient was brought to the emergency room with a decreased level of responsiveness for the past hour. On initial assessment, the patient was stuporous and diaphoretic, and had frothy oral secretions and increased work of breathing. He was immediately transferred to the RED zone, connected to continuous cardiac monitoring, and two large-bore intravenous (IV) cannulas were secured. The primary survey is shown in Table 1.

**Table 1:** Primary survey RA: room air; RR: respiratory rate; IV: intravenous; B/L: bilateral; CVS: cardiovascular system; CBG: capillary blood glucose; GCS: Glasgow Coma Scale; NS: normal saline

Parameter	Findings	Interventions
Airway	Threatened	Oral suctioning done
	Frothy secretions present	
Breathing	Tachypneic, dyspneic	In view of impending respiratory failure, patient was intubated and connected to mechanical ventilator
	B/L air entry, B/L crackles	
	SpO_2_: 65% on RA	
	RR: 30/minute	
Circulation	Heart rate: 180/minute	IV fluid 0.9% normal saline at 100 mL/hour started
	Blood pressure: 120/90 mmHg	
	Peripheral pulses felt	
	CVS: S1S2	
Disability	CBG: 150 mg/dL	-
	GCS: E2VTM4	
	B/L pupil 2 mm equal and reactive to light	
Exposure	Temperature: 108°F	Inj. paracetamol 1-gm IV infusion followed by IV fluid NS 1,000 mL bolus
	Involuntary urination and defecation	External cooling measures (ice packs) were done

Primary survey adjuncts

The arterial blood gas analysis indicates high anion gap metabolic acidosis with an increased lactate level of 5 mmol/L. The ECG shows sinus tachycardia. A Ryle's tube was inserted, and gastric lavage was performed using cold saline, which revealed food particles. Additionally, a Foley catheter was inserted, resulting in a urine output of 300 mL, which was noticeably dark in color. Upon reassessment, he was hemodynamically stabilized; his temperature decreased from 108°F to 103°F, and his heart rate decreased from 180 to 110 bpm. The secondary survey is shown in Table 2.

**Table 2:** Secondary survey PMH: past medical history

Secondary survey	
Signs and symptoms	History of fever for three days, for which the patient took over-the-counter medication
	History of breathing difficulty since morning, with one episode of vomiting
	History of recent travel to Pune
	No other significant history
Allergic history	Nil
Medications and PMH	Over-the-counter fever medications for the past two days
	No known significant comorbidities
	Nonalcoholic and nonsmoker
Last meal	Two hours before the presentation
Events leading to illness	Not known

Secondary survey adjuncts

High-resolution CT of the thorax suggests aspiration pneumonia. CT of the brain shows no significant abnormalities. USG of the abdomen reveals mildly raised cortical echoes and mild gallbladder edema. An echocardiogram indicates adequate left ventricular function.

Based on the patient’s history, clinical presentation, and initial investigations, our working diagnosis was sepsis with metabolic encephalopathy. However, given the presentation of hyperpyrexia and altered mental status, thyrotoxic crisis was also kept as a strong differential. Investigations were sent accordingly. In view of suspected sepsis, the sepsis one-hour bundle was promptly initiated, and empirical antibiotics were started. Since thyrotoxic crisis remained a possibility, we assessed the patient using the Burch-Wartofsky Point Scale, which yielded a score of 90, strongly suggestive of a thyroid storm. Supportive care, including IV fluids, oxygen supplementation, antipyretics, and external cooling measures, had already been initiated based on his initial presentation.

The patient was hemodynamically stabilized in the Emergency Department, with working differentials including sepsis with metabolic encephalopathy and thyrotoxic crisis. He was then transferred to the intensive care unit (ICU) for further management. Based on the clinical presentation and supported by laboratory findings (as shown in Table 3), a diagnosis of thyrotoxic crisis triggered by sepsis, complicated by multiorgan dysfunction and DIC, was made.

**Table 3:** Laboratory parameters ^*^Abnormal laboratory parameters T3: triiodothyronine; T4: thyroxine; TSH: thyroid stimulating hormone; CBC: complete blood count; WBC: white blood cells; aPTT: activated partial thromboplastin time; PT: prothrombin time; LDH: lactate dehydrogenase; LFT: liver function test; SGOT: serum glutamic oxaloacetic transaminase; SGPT: serum glutamic pyruvic transaminase; ALK: alkaline phosphatase; GGT: gamma glutamyl transferase; Sr: serum; WNL: within normal limit; C3 and C4: complement

Workup	Day 1	Day 3	Day 5	Day 7	Day 9	Day 11	Day 13	Day 47
Free T3 (pg/mL)	14.6^*^	5.67^*^	11.5^*^	8.94^*^	8.38^*^	-	7.81^*^	3.57^*^
Free T4 (pg/mL)	6.38^*^	4.99^*^	4.58^*^	3.75^*^	4.47^*^	-	3.26^*^	0.89^*^
TSH (mIU/L)	<0.01^*^	-	-	-	-	-	-	<0.01^*^
CBC								
WBC (cells/mm^3^)	11.89^*^	10.01^*^	4.86	6.24	6.76	8.72	5.30	-
Polymorphs (%)	93.5^*^	84.9^*^	80.6^*^	69.4	69.2	72.7	32.2	-
Hemoglobin (g/dL)	14.4	11.8^*^	11.4^*^	10.1^*^	9.7^*^	10.7^*^	13.4	-
Platelet (lakhs)	68,000^*^	42,000^*^	60,000^*^	118,000^*^	144,000^*^	-	414,000	-
D-dimer (ng/mL)	-	9,610^*^	-	-	-	-	-	-
APTT (25-35 seconds)	42.2^*^	37^*^	32.8	-	-	-	-	-
PT (12-15 seconds)	24.6^*^	29.8^*^	15.2^*^	13.8	-	-	-	-
Fibrinogen (mg/dL)	-	223	-	-	-	-	-	-
Procalcitonin (ng/mL)	1.7^*^	-	0.46	-	0.17	-	-	-
LDH (U/L)	1,000^*^	545^*^	-	-	-	-	-	-
Ferritin (ng/mL)	-	3,430^*^	-	-	-	-	-	-
Ionized calcium (mg/dL)	-	4.28	-	-	-	-	-	-
Sr. cortisol (mcg/dL)	-	16.9	-	23.7^*^	-	-	9.54	-
Sr. creatinine (mg/dL)	0.8	2.6^*^	1.0	0.7	0.5	-	0.5	-
Sr. urea (mg/dL)	19	98^*^	127^*^	97^*^	86^*^	-	-	-
Sr. uric acid (mg/dL)	8.9^*^	-	-	-	-	-	-	-
Electrolytes								
Sodium (mmol/L)	136	136	147^*^	149^*^	146^*^	-	-	-
Potassium (mmol/L)	3.5	4.3	3.4^*^	3.2^*^	2.9^*^	-	-	-
Chloride (mEq/L)	110^*^	111^*^	117^*^	119^*^	112^*^	-	-	-
Bicarbonate (mEq/L)	15^*^	15^*^	22^*^	27	28	-	-	-
Phosphorus (mg/dL)	3.0	-	-	-	-	-	-	-
Magnesium (mg/dL)	1.7	-	-	-	-	-	-	-
LFT								
SGOT (U/L)	1,602^*^	1,562^*^	527^*^	180^*^	96^*^	-	-	-
SGPT (U/L)	1,016^*^	1,912^*^	1,230^*^	832^*^	532^*^	-	-	-
ALK (U/L)	175^*^	146^*^	147^*^	120	122	-	-	-
GGT (U/L)	62^*^	41	180	214	213	-	-	-
Albumin (g/dL)	3.5	2.6^*^	3.1^*^	3.1^*^	3.1^*^	-	-	-
Bilirubin								
Total (mg/dL)	2.2^*^	3.2^*^	4.1^*^	3.2^*^	3.2^*^	-	-	-
Direct (mg/dL)	1.8^*^	2.9^*^	3.5^*^	2.7^*^	2.6^*^	-	-	-
Indirect (mg/dL)	0.4	0.3	0.6	0.5	0.6	-	-	-
Sr. ammonia (µmol/L)	-	-	55^*^	-	-	-	-	-
Sr. cholinesterase (U/L)	5,768^*^	-	-	-	-	-	-	-
Sr. creatine kinase (U/L)	398^*^	-	-	-	-	-	-	-
Triglycerides (mg/dL)	-	426^*^	-	-	-	-	-	-
Urine analysis: WNL; Sugar eyetone: WNL; C3 and C4: WNL; HbA1C: 5.5%; Blood culture: sterile; Urine culture: sterile								

**Figure 1:**
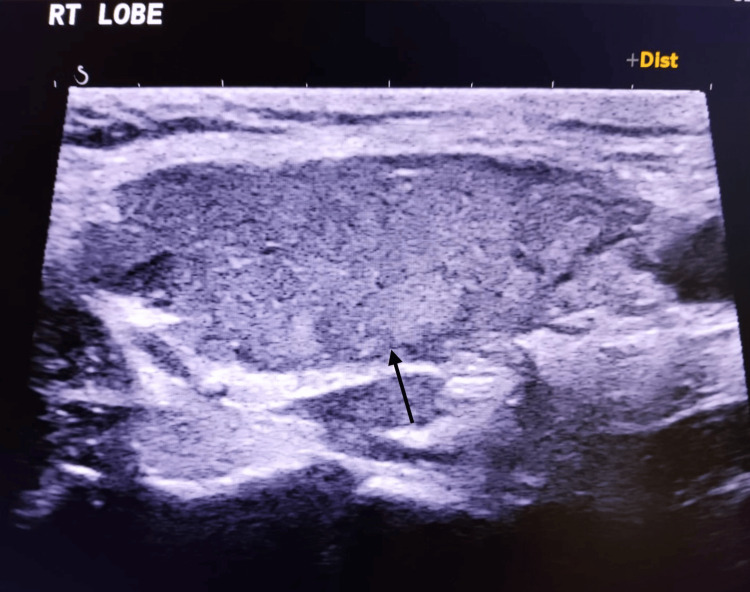
Right lobe of the thyroid gland showing a nonnodular heterogeneous pattern RT: right

**Figure 2:**
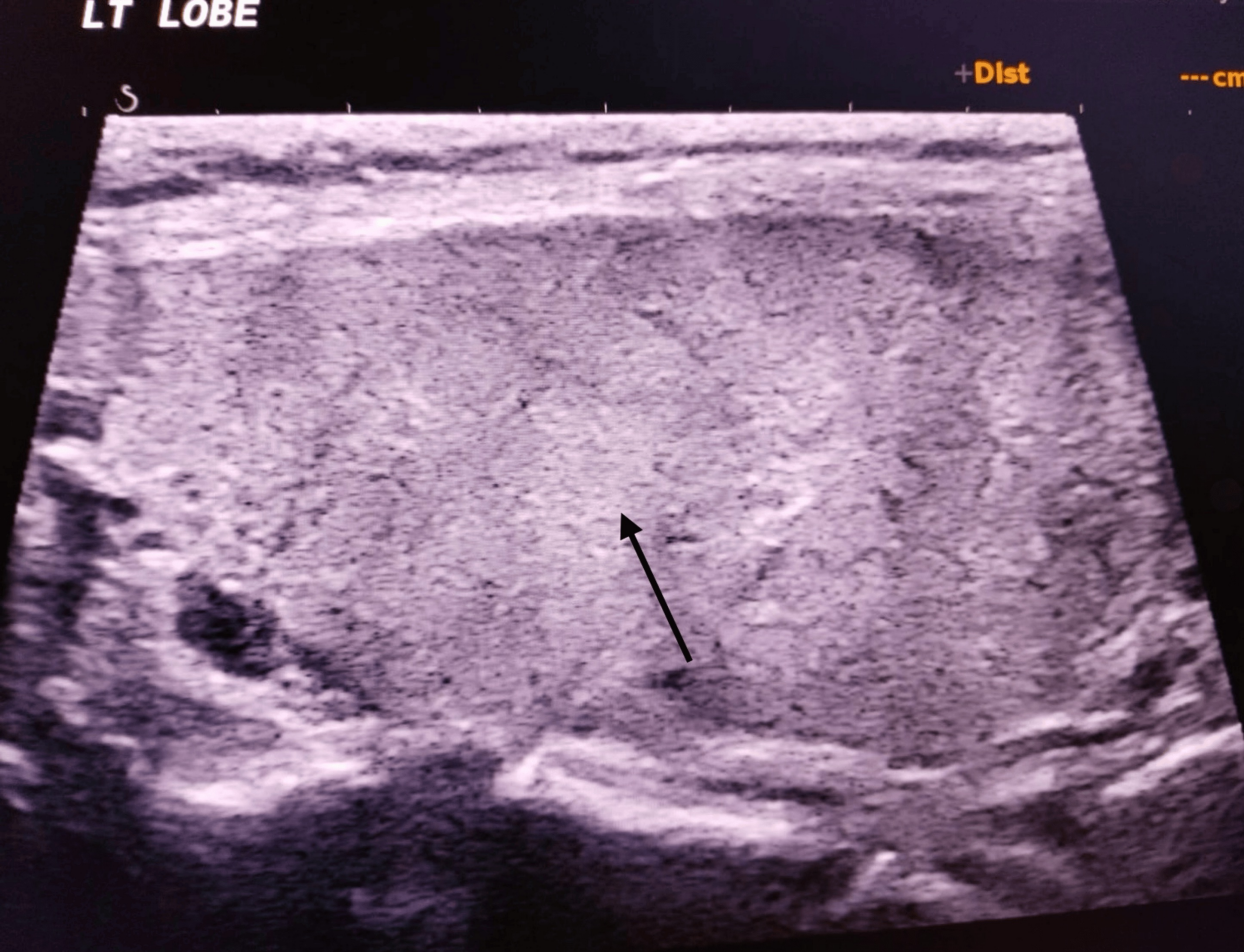
Left lobe of thyroid gland showing a nonnodular heterogeneous pattern LT: left

**Figure 3:**
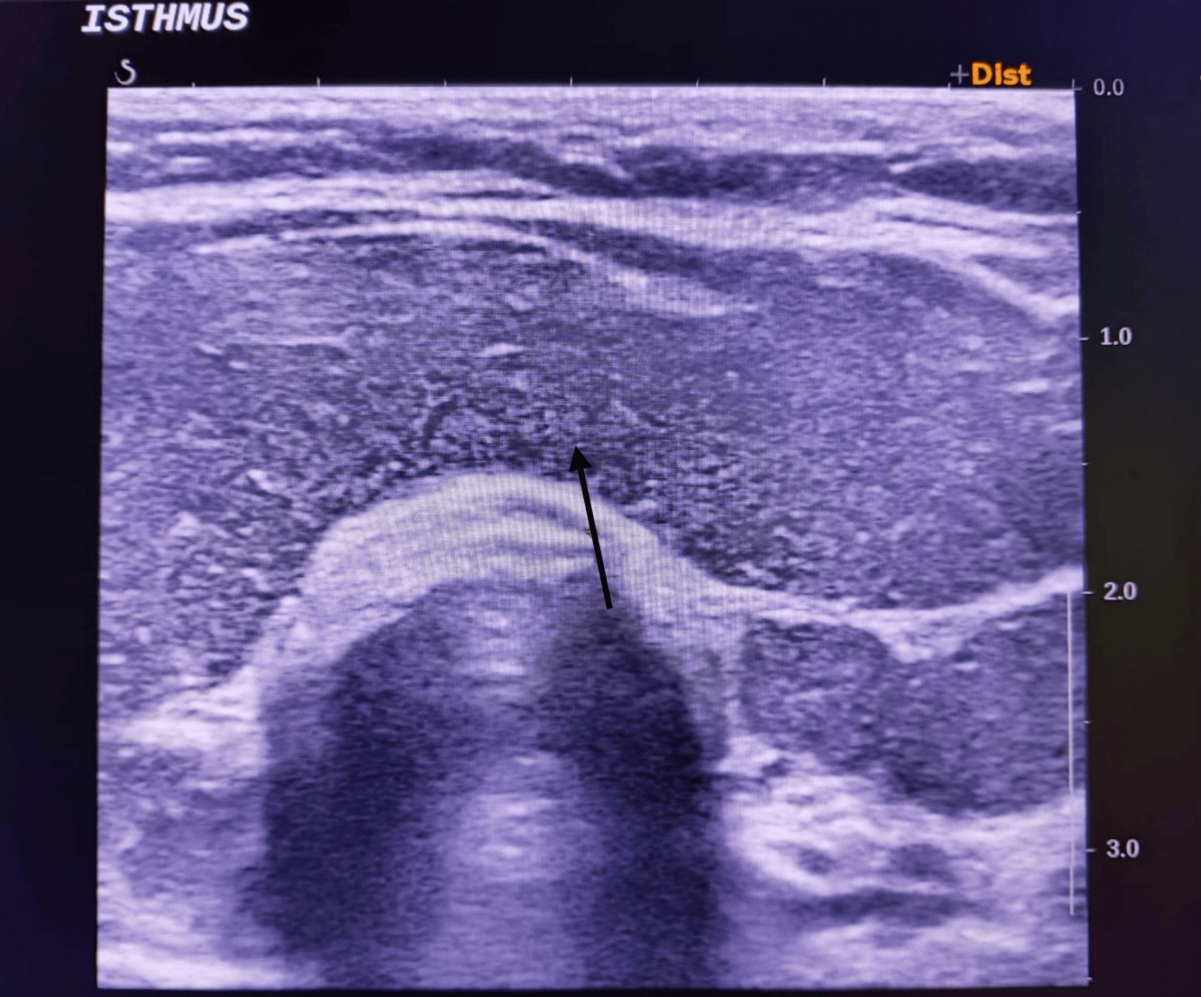
Isthmus of the thyroid gland showing a nonnodular heterogeneous pattern

The patient was then started on beta-blockers, appropriate thionamide, steroids, cholestyramine, and antibiotics, while supportive care was continued. Within eight hours of ICU admission, his Glasgow Coma Scale improved to E4VTM5, and his temperature settled to 100°F. Given the involvement of the hepatic, renal, endocrine, and cardiac systems, relevant specialty teams were involved, and he was managed accordingly. USG thyroid was done, which showed features of a nonnodular heterogeneous pattern (as shown in Figures 1-3), and a Tc-99m thyroid scan was advised to be performed later after clinical stabilization of the patient. He continued to show steady improvement in the ICU and was eventually extubated and transferred to the step-down unit for ongoing cardiac monitoring.

Following transfer from the ICU, the patient was monitored in the step-down unit and subsequently in the ward for over seven days. During his stay, the Tc-99m thyroid scan was performed, which showed features of thyromegaly with diffuse high tracer uptake consistent with hyperfunctioning thyroid. Throughout his stay, he remained comfortable, and clinically and hemodynamically stable, with no new complaints. He was discharged in good condition with instructions to follow up in the outpatient clinic.

## Discussion

The term “thyrotoxic crisis” was first described as “the crisis of exophthalmic goiter” in 1928, which was universally fatal without treatment [3]. Although it is a rare and life-threatening complication of hyperthyroidism, its mortality remains high without timely intervention, and morbidity increases with delayed treatment [3,7]. Therefore, early recognition and prompt intensive management of thyroid storm are essential to reduce both mortality and morbidity [7]. Based on the etiology of thyrotoxic crisis, Graves’ disease accounts for approximately 71.4% of cases, followed by toxic multinodular goiter, which accounts for 14.3% [3]. In view of the complications associated with thyrotoxic crisis, these include arrhythmias, cardiac failure, liver injury, atrial fibrillation, seizures, and coma [1].

According to Indian estimates, the cardiovascular system (94.3%), including tachycardia (80%) as the most common presentation, followed by heart failure (60%) and atrial fibrillation (40%), and the gastrointestinal-hepatic system (88.6%), which includes moderate symptoms in 74.3% and severe symptoms in 14.3% (according to the Burch-Wartofsky Point Scale), are the most common presenting features [3,8]. Central nervous system involvement was observed in only 42.3% of patients, of which mild symptoms were seen in 25.7%, moderate in 16.6%, and none exhibited severe symptoms according to the Burch-Wartofsky Point Scale [3]. Thermoregulatory dysfunction was found in only 22% of patients. The mean age at presentation is 44 ± 10.2 years, with a female preponderance of 71.4% [3]. In terms of diagnosis, the Burch-Wartofsky Point Scale is a widely used diagnostic tool for thyrotoxic crisis, with a reported positive predictive value of 75% and a negative predictive value of 100% [9].

DIC is a hypercoagulable state in which both micro- and macrovessels become clogged by clots, leading to multiorgan dysfunction syndrome [10-12]. The most common cause of DIC is sepsis; however, it has multiple other causes, which also include liver injury [10]. Although DIC occurs as a rare complication of thyrotoxic crisis, it is important to recognize it. While there are case reports mentioning DIC secondary to thyrotoxic crisis, the association between DIC and thyrotoxicosis is yet to be well elucidated [5,6,12-14]. Certain mechanisms have been proposed to delineate this association, in which thyroid storm causes a systemic inflammatory response syndrome (SIRS) [15] through an exaggerated release of proinflammatory cytokines [5,8,13]. In addition, thyroxine stimulates the production of interleukin (IL)-1, which, in turn, activates platelets and increases the production of von Willebrand factor, IL-6 and IL-8, and tissue factor (TF), all of which collectively create a prothrombotic environment [5,6,13].

Sepsis also triggers an SIRS, marked by the release of proinflammatory cytokines such as IL-6 and tumor necrosis factor-alpha. These cytokines upregulate TF expression on monocytes and endothelial cells, activating the extrinsic coagulation pathway and contributing to the development of DIC [16].

In our case, the patient was a man who presented with hyperpyrexia, altered mental status, tachycardia, and vomiting, without any known comorbidities. While all clinical features correlated with thyrotoxic crisis, the key feature, hyperpyrexia, made us think of thyrotoxic crisis. However, due to the history of fever in the preceding days, our primary diagnosis was sepsis, with thyrotoxic crisis considered as a differential. By keeping thyrotoxic crisis in mind and identifying it early through clinical criteria along with supporting laboratory findings, we were able to successfully manage the patient without morbidity. In view of the DIC, in this case, it could have occurred secondary to liver injury, as evidenced by laboratory findings. It could also have occurred secondary to sepsis as well as thyrotoxic crisis. Although there were multiple possible causes for DIC, its rapid onset and resolution following treatment with thionamides and antibiotics suggest that it may have been a complication of both sepsis and thyrotoxic crisis. Since sepsis and thyrotoxic crisis contribute to the development of DIC through a similar inflammatory pathway, it is possible that sepsis exacerbates the process of DIC or vice versa.

This case highlights the importance of emergency physicians, who are often the first point of contact, to consider endocrine emergencies such as thyrotoxic crisis in their differential diagnosis when encountering patients with similar presentations to improve patient outcomes. Even though it is unclear whether the exact cause of DIC is sepsis or thyrotoxic crisis, it is always important to recognize it as a potential complication of thyrotoxic crisis.

## Conclusions

In conclusion, we report a case of thyrotoxic crisis triggered by sepsis, complicated by DIC and multiorgan dysfunction syndrome, who was admitted, treated, and discharged home successfully without any morbidities. Since timely identification and intervention are crucial to prevent adverse outcomes, early recognition by emergency physicians, as the first point of contact, of thyrotoxic crisis as a differential diagnosis for hyperpyrexia is essential. Even though DIC is a rare complication and there are case reports that mentions an association between thyrotoxic crisis and DIC, the relationship remains unclear, it should still be recognized as a potential complication to ensure appropriate management and improve patient prognosis.
